# An IoT-Based Framework for Automated Assessing and Reporting of Light Sensitivities in Children with Autism Spectrum Disorder

**DOI:** 10.3390/s24227184

**Published:** 2024-11-09

**Authors:** Dundi Umamaheswara Reddy, Kanaparthi V. Phani Kumar, Bandaru Ramakrishna, Ganapathy Sankar Umaiorubagam

**Affiliations:** 1 Diagnostics, Therapeutics and Assistive Devices Laboratory, Department of Electronics and Communication Engineering, Faculty of Engineering and Technology, SRM Institute of Science and Technology, Kattankulathur 603203, Tamil Nadu, India; dr9109@srmist.edu.in; 2 School of Electronics Engineering (SENSE), VIT-AP University, Vijayawada 522241, Andhra Pradesh, India; ramakrishna.bandaru@vitap.ac.in; 3 SRM College of Occupational Therapy, SRM Institute of Science and Technology, Kattankulathur 603203, Tamil Nadu, India; ganapatu@srmist.edu.in

**Keywords:** autism spectrum disorder, light sensitivities, internet of things, computer vision, monitoring

## Abstract

Identification of light sensitivities, manifesting either as hyper-sensitive (over-stimulating) or hypo-sensitive (under-stimulating) in children with autism spectrum disorder (ASD), is crucial for the development of personalized sensory environments and therapeutic strategies. Traditional methods for identifying light sensitivities often depend on subjective assessments and manual video coding methods, which are time-consuming, and very keen observations are required to capture the diverse sensory responses of children with ASD. This can lead to challenges for clinical practitioners in addressing individual sensory needs effectively. The primary objective of this work is to develop an automated system using Internet of Things (IoT), computer vision, and data mining techniques for assessing visual sensitivities specifically associated with light (color and illumination). For this purpose, an Internet of Things (IoT)-based light sensitivities assessing system (IoT-LSAS) was designed and developed using a visual stimulating device, a bubble tube (BT). The IoT-LSAS integrates various electronic modules for (i) generating colored visual stimuli with different illumination levels and (ii) capturing images to identify children’s emotional responses during sensory stimulation sessions. The system is designed to operate in two different modes: a child control mode (CCM) and a system control mode (SCM). Each mode uses a distinct approach for assessing light sensitivities, where CCM uses a preference-based approach, and SCM uses an emotional response tracking approach. The system was tested on a sample of 20 children with ASD, and the results showed that the IoT-LSAS effectively identified light sensitivities, with a 95% agreement rate in the CCM and a 90% agreement rate in the SCM when compared to the practitioner’s assessment report. These findings suggest that the IoT-LSAS can be used as an alternative to traditional assessment methods for diagnosing light sensitivities in children with ASD, significantly reducing the practitioner’s time required for diagnosis.

## 1. Introduction

Autism spectrum disorder (ASD) is a neurodevelopmental disorder that affects communication, cognitive abilities, and social skills in individuals [1,2,3]. Sensory sensitivities are key features for diagnosing ASD, where the central nervous system does not process or respond to received sensory stimuli in the desired way [4,5]. In 2013, the American Psychiatric Association highlighted sensory sensitivities as important factors for diagnosing ASD [1]. These sensitivities may be observed in any sensory system, either auditory, visual, tactile, or olfactory [6,7]. Among these, visual or light sensitivity is a common sensory issue in children with ASD, which may lead to behavioral and learning difficulties [8,9]. The literature highlights that light and visual sensory sensitivity is reported by up to 75% of individuals with ASD and may exhibit varying degrees of sensitivity, such as hyper- or hypo-sensitivity. For example, a child who is hyper-sensitive often avoids bright lights, and they may become frightened by sudden flashes, while hypo-sensitive children are typically drawn to light, showing fascination with bright lights and their reflections [10,11,12,13,14]. According to the existing literature, visual sensitivities in individuals with ASD are frequently associated with certain colors or variations in illumination levels [8,15,16]. Identifying how children with ASD respond to sensory input is crucial for clinicians. This understanding helps them uncover the reasons behind the child’s challenging behaviors and enables them to create adaptive environments tailored to each child’s individual needs. Snoezelen rooms, also known as sensory rooms (SRs) or multi-sensory environments (MSEs), are specially designed to better understand and analyze the impact of sensory stimulation on individuals with neurodevelopmental disabilities under different conditions. These rooms include devices such as bubble tubes, wall panels, optical fibers, mirror balls, etc., to stimulate the senses of sight, touch, hearing, and smell [17,18,19,20]. Traditionally, sensory processing patterns in children have been identified using various assessment methods, including (i) collecting data through questionnaires and rating scales [21,22] and (ii) observing and annotating video recordings [23,24]. In the first approach, clinical practitioners gather information from caregivers or parents by asking about the child’s responses to different sensory stimuli faced in daily life. In the video observation method, clinicians record the child’s activities and manually annotate the videos to analyze their sensory responses. However, traditional subjective assessment methods may not capture the full range of sensory responses experienced by children, as they depend on information gathered from parents or caregivers. Additionally, video annotation methods, while potentially more detailed, are often time-consuming and require significant effort for accurate analysis. It is challenging for clinical practitioners to identify immediate over-stimulating and under-stimulating sensitivity behaviors exhibited by children through manual observations in sensory rooms. This limitation raises the need for automated methods that can provide more objective and comprehensive evaluations of sensory processing patterns in children with ASD.

Recent technological advancements have introduced novel approaches to assessing sensory challenges in individuals with ASD. Researchers have explored the use of sensors to objectively measure physiological responses to sensory stimuli, offering valuable insights into individual sensory profiles. In a study [25], researchers utilized force, touch, and RGB-D sensors to measure the functions of children with ASD in different test scenarios, transmitting the collected data to devices for continuous monitoring and analysis. Similarly, in another study [26], a wearable tactile sleeve was developed that senses virtual experiences, simulating the sensation of being touched for hypersensitive individuals with ASD. Additionally, there is a study where an ACHI prototype was proposed as an assistive-technology-based companion, which is capable of detecting sensory information through electronic sensors specifically tailored for hypersensitive individuals with ASD [27]. There are also studies that highlight the importance of automated assessing and reporting methods in health care [28,29]. This shows that technological innovations are paving the way for more objective, precise, and individualized assessments of sensory processing challenges in individuals with ASD. By utilizing advanced sensors and wearable devices, these technologies allow for the real-time monitoring of physiological responses to sensory stimuli, providing continuous and detailed data on sensory sensitivities. This objective approach not only enhances the understanding of each individual’s unique sensory profile but also supports the development of personalized interventions that can more effectively address their specific needs. The use of such technology represents a significant shift from traditional subjective assessment methods, offering a more comprehensive and accurate way to assess and manage sensory sensitivities in ASD.

**Figure 1:**
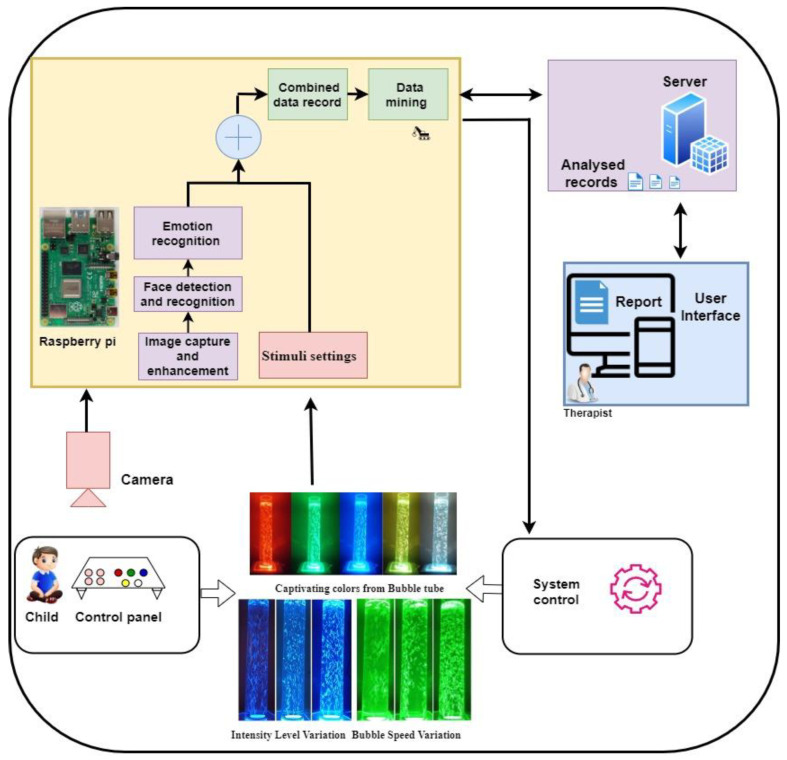
Architecture of IoT LSAS.

However, in MSEs or SRs, the clinical practitioners use video observation and manual coding methods for assessing sensory stimuli preferences and emotional responses exhibited by children with ASD. Though it is the most used method, the identification of sensory responses relies on careful observations and manual analysis. An assistive assessment system for clinical practitioners that automatically captures sensory preferences and responses of children with ASD is more beneficial. The present work focuses on the design and development of an automated system that is capable of providing more comprehensive and objective assessments of each child’s sensory preferences and emotional responses for assessing light sensitivities. An IoT-enabled light sensitivity assessing system using IoT, computer vision, and data mining techniques is proposed, which is shown in Figure 1. The proposed system uses the bubble tube (BT), which is popular for its captivating sensory stimulation in MSEs and provides colorful visual experiences [30,31,32,33]. Further, the overview, design, development, and evaluation results of the proposed IoT-LSAS are discussed in the following sections.

## 2. Materials and Methods

### 2.1. Overview of IoT-LSAS

The proposed IoT-LSAS uses computer vision and IoT technologies to monitor and assess light sensitivities in children with ASD. For visual stimuli, a dual control BT is designed and developed, which can be operated in two modes: the child control mode (CCM) and the system control mode (SCM). In the CCM, the child can generate his/her preferred stimuli with the help of control panel by selecting colors and illumination levels. In the SCM, the child will not have control over the stimuli; the system generates colors in automated way with different illumination levels in fixed intervals to observe and log emotional responses of child for each combination. The Raspberry Pi module, which is a mini-computer, collects preference data and emotional response data in respective modes of operation. The collected data are further saved with child identification number (ID) who is interacting with bubble tube. Each saved file includes data records with date and time logs, colors, illumination levels, durations, or emotional responses. These data are further analyzed with data mining techniques for identifying frequent preferred patterns and associations. From this information, further analysis is carried out in server to generate detailed reports on color and illumination sensitivities based on support values given by data mining algorithm.

The design and development of IoT-LSAS are conceptualized into four major components: (i) fabrication of the bubble tube, control panel, and integration of the required electronic circuitry (hardware), (ii) implementation of the dual mode operation (child and system control modes), (iii) implementation of emotion detecting and data logging modules, and (iv) data mining and classification. Each of these components is elaborated further in following sections.

### 2.2. Fabrication Process of Bubble Tube, Control Panel and Their Features

**Figure 2:**
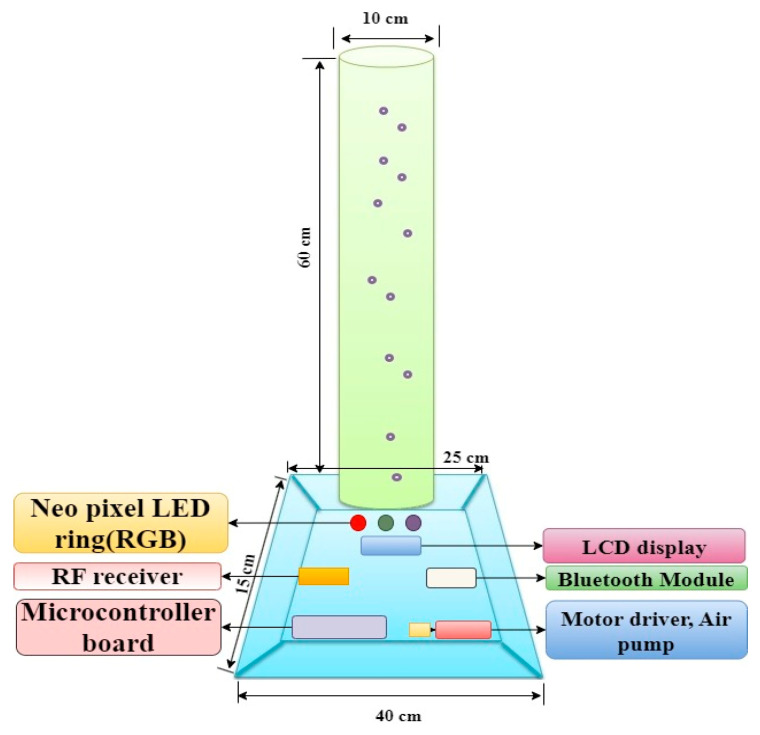
Design of bubble tube used to generate colored visual stimuli.

**Figure 3:**
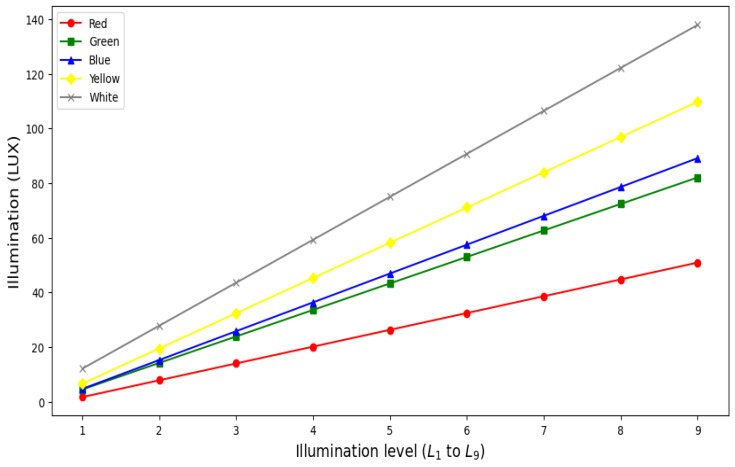
Illumination for different intensity levels measured using LUX meter.

For generating various colors with different illumination levels, a BT is fabricated using a 5 mm thick transparent acrylic tube, as per the design shown in Figure 2. The acrylic tube is filled with distilled water and is placed on a specially designed wooden box. The dimensions of the acrylic vertical tube (nearly 60 cm in length and 10 cm outer diameter) are adopted from guidelines provided by the Ministry of Health & Family Welfare Government of India’s operational guidelines, May 2014 [34]. Initially, a 5 mm thick circular grove with 10 cm diameter is made on the top surface of the wooden box to hold the bubble tube and also to keep the Neo pixel RGB LED ring at the bottom of the tube. The Neopixel LED ring is used for multicolor illumination and is interfaced with the microcontroller for color generation. Secondly, a circular airflow tube (0.5 cm diameter) with multiple holes is fixed at the bottom of the bubble tube. Using the air pump and its driving circuit, the controlled flow of bubbles can be introduced into the filled distilled water. The other electronic components, such as the RF module, microcontroller unit, motor driver, and air pump, are placed inside the wooden box. A 9V-2A DC adaptor is used to power all the electronic components. The BT can generate five distinct colors (red, green, blue, yellow, and white) with three intensity levels (low, medium, and high). These colors were carefully chosen to offer a varied visual palette, considering the participants’ varying light sensitivities. By using these primary (red, green, blue) and secondary (yellow, white) colors, we can create a range of illumination levels from low to high intensity. For example, using red and green, we can generate low illumination levels, and with blue, moderate illumination levels can be generated. While using yellow and white, higher illumination levels can be generated. The BT will also generate three levels of bubble speed variations (low, medium, and high). For each color intensity level combined with bubble speed level, the BT offers total of nine illumination levels (L1–L9) of intensity variation, which are illustrated in Figure 3. For example, illumination level L1 corresponds to color intensity level I1 combined with bubble speed level B1, while L9 represents the highest combination of color intensity level I3 and bubble speed level B3. The intensity levels shown are measured using a lux meter at a one-meter distance from the bubble tube’s surface. It is important to note that the intensity levels are measured at a distance of 1 m from the bubble tube’s surface. The inverse square law of illumination states that the intensity of light decreases in proportion to the square of the distance from the light source, meaning that as you move closer, the brightness increases. At half a meter from the bubble tube, the illumination is four times higher, and near the surface of the BT, the illumination is approximately eight times the measured value at 1 m. The bubbles feature is included to attract participants for their active exploration and to maintain maximum engagement.

### 2.3. Design of Control Panel (CP)

**Figure 4:**
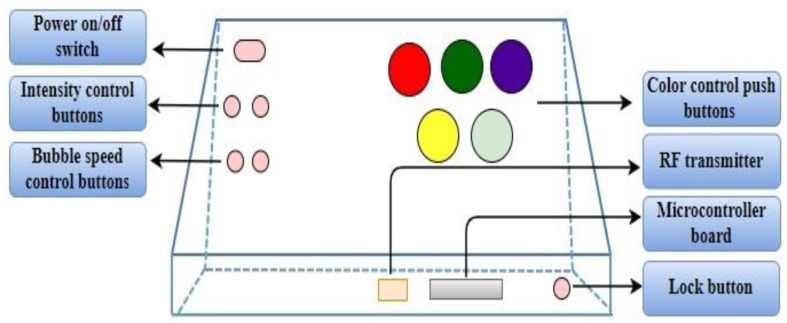
Customized control panel for active intervention, consisting of five color control illuminated push buttons for selection of colored illumination and two sets of push buttons for intensity and bubble speed control.

The CP is fabricated as per the design shown in Figure 4 for children to control visual stimuli generated by BT. CP consists of five illuminated push buttons for color selection, which allows participants to choose a variety of colors as per their interests. Additionally, four push buttons are provided, of which two are for controlling the intensity levels and the other two push buttons to regulate the bubble speed. The amusement arcade push buttons were used to provide ease of control and durability. The microcontroller unit is placed inside the panel to process the user input from the push buttons and to transmit the corresponding data to BT. The control panel establishes wireless communication using RF transmitter and receiver, allowing immediate adjustments of the sensory stimulating parameters. All the electronics are integrated within a wooden frame to ensure a robust and durable interface specifically designed for children. The choice of a wooden frame was made considering the sturdiness and capacity to withstand repeated interaction and manipulation by children and also ensuring its durability and consistency throughout sessions.

### 2.4. Implementation of the Dual Mode Operation (Child and System Control Modes)

The proposed IoT-LSAS can be operated in two modes: the child control and system control modes. These two modes act as a key feature for assessing visual sensory sensitivities in children.

#### 2.4.1. Child Control Mode (CCM)

CCM is one where child interacts directly with the BT using the CP for generating preferred stimuli. In CCM, the focus is on understanding the child’s natural preferences for colors and illumination levels. In this mode, the child is given control over the bubble tube, allowing them to select colors and adjust illumination levels. The system records these interactions, capturing data such as the selected colors, light intensities, and the duration of preference. This mode provides information about the child’s favored visual stimuli, which helps to identify their preferences and avoid stimuli.

#### 2.4.2. System Control Mode (SCM)

In SCM, the system takes full control of BT, automatically generating visual stimuli at fixed intervals. The purpose of SCM is to assess how the child reacts to different visual stimuli combinations The system systematically varies the color and illumination levels at fixed intervals of 5 s to observe and capture the child’s emotional responses to these controlled conditions. The interval of 5 s is chosen to allow the system to capture the child’s emotional responses to generated stimuli. Using image processing and deep learning techniques, the system analyzes the child’s facial expressions to identify emotions like joy, surprise, neutrality, sadness, or fear. This mode is crucial for identifying whether certain light conditions cause hyper-sensitivity, hypo-sensitivity, or a neutral response.

Both modes provide a comprehensive understanding of a child’s sensory processing capabilities. The CCM captures the child’s natural preferences and reactions, whereas the SCM tests emotional reactions under controlled conditions to further analyze and classify sensory sensitivities.

### 2.5. Implementation of Facial Recognition, Emotion Detection, and Data Mining Modules

Required hardware and software modules are developed for recognizing and classifying children’s emotional responses to visual stimuli and mining the logged data records during child interaction with BT. The developed software modules are deployed to Raspberry Pi 4 (8GB RAM), which is a mini-computer capable of running deep learning models using Tensor Flow Version 2.9. The Logitech HD cam (Logitech, Delhi, India) is interfaced with Raspberry Pi4 to capture images of children during the interaction sessions.

#### 2.5.1. Child Face Recognition and Emotions Detecting Module

The child may exhibit any one of the facial emotions like joy, surprise, anger, fear, sadness, and neutrality based on the adaptive and defensive stimuli illuminated by the BT. The face recognition and emotions detecting modules are used to detect facial emotions exhibited by children. Due to the varying lighting conditions and intensity fluctuations from the bubble tube, the captured images can exhibit inconsistent brightness and contrast levels. So, before passing the captured images to facial recognition module, the images are initially enhanced using gamma correction and upscaling methods. Different gamma values are used to enhance the images effectively.

Gamma = 0.5: To brighten the image captured in a low-intensity environment, increasing visibility in darker areas.

Gamma = 0.8: Slightly brightens the image, enhancing mid-tones without overly lightening highlights.

Gamma = 1.5: To darken the image by reducing brightness in overly bright images captured during high-intensity stimuli from BT.

The enhanced image is then processed by the dlib facial recognition library for child face detection and recognition. Dlib is a widely used toolkit for facial detection and recognition, which includes high-performance machine learning algorithms. The process begins with detecting the facial landmarks using dlib’s pre-trained model, which identifies the key facial features such as eyes, nose, and mouth. Once the child’s face is detected from image, the dlib’s facial recognition algorithm compares the detected face with known faces in the database, which is saved previously to identify the child. Following this, the image is passed through an emotion recognition model for facial emotion recognition. Further, the training and testing of emotion recognition model used in this work are discussed in following section.

#### 2.5.2. Training Dataset for Emotion Recognition Model Generation

**Figure 5:**
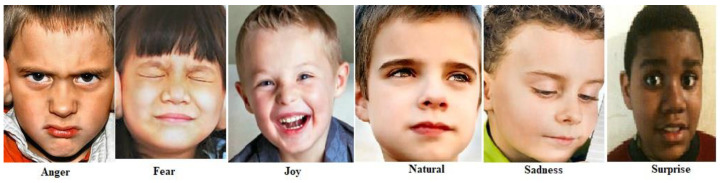
Six distinct facial emotions of children with ASD (images shown are taken from openly available autism children emotions dataset from kaggle).

**Figure 6:**
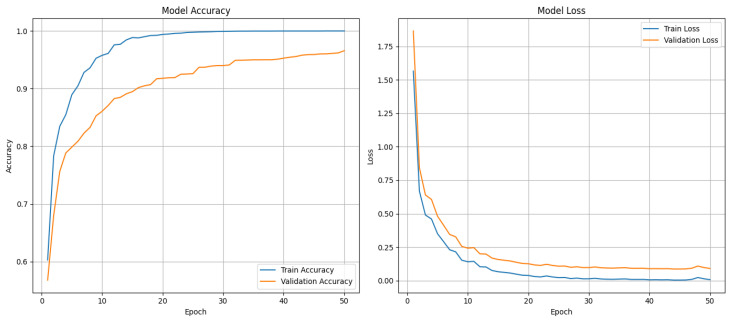
Plot showing the performance of the trained model for 50 epochs.

**Figure 7:**
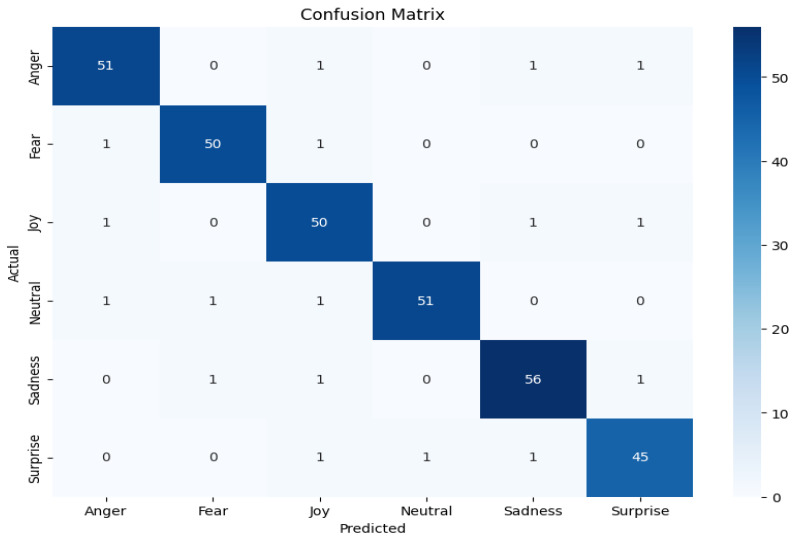
Confusion matrix showing the correct and wrong predictions for each class.

This work uses a combined dataset, which is prepared by adding custom-made dataset of autistic children with openly available autistic children’s emotions dataset from kaggle. The dataset from kaggle includes 758 images divided into 6 classes: anger (67 images), fear (30 images), joy (350 images), neutrality (48 images), sadness (200 images), and surprise (63 images). This dataset was combined with the custom-made data of autistic children coming to autism center to equalize the 6 emotion classes. This enhancement was completed to achieve better accuracy by providing a more balanced and extensive collection of images for training across all the classes. The six primary emotions used are shown in Figure 5. These emotions, such as anger, fear, joy, neutrality, sadness, and surprise, were selected based on established psychological theories of emotion and their relevance in assessing emotional responses in children with ASD. Each emotion is shown with corresponding facial expressions to aid in understanding how they manifest visually. The generated new dataset comprises total of 2114 images divided into 6 classes: anger (355 images), fear (345 images), joy (350 images), neutrality (356 images), sadness (390 images), and surprise (318 images). Of the total set of images, 85% are used for training, and 15% of images are used for validation. This combined dataset was used to train a facial emotion recognition model using Google’s Teachable Machine, which is a web-based tool that simplifies the process of creating deep learning models. Teachable Machine allows users to train models by providing a user interface for uploading datasets, defining categories, and training the model. A set of training experiments was conducted by adjusting key parameters such as batch size, learning rate, and epochs to optimize the performance of the neural network. Through these experiments, it was observed that a learning rate of 0.001, 50 epochs, and a batch size of 32 generated the best performance model with training accuracy of 99.86% and validation accuracy of 94.69%. The performance of the trained model for 50 epochs is shown in Figure 6. The confusion matrix showing the correct and wrong predictions for each class is shown in Figure 7. The trained model was then evaluated using the testing set by uploading images to the interface to ensure its accuracy and reliability in recognizing the six defined emotions and that all the emotions are recognized correctly.

### 2.6. Logged Data Records and Their Attributes

**Figure 8:**
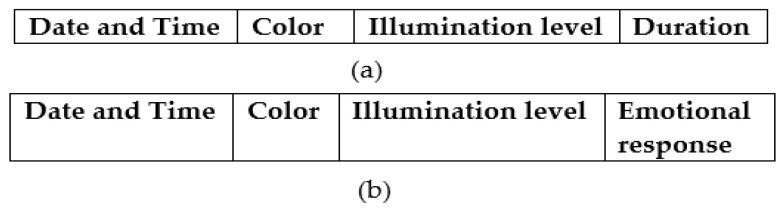
Logged data record and respective fields in two modes: (**a**) CCM and (**b**) SCM.

Each child’s interactions with BT in CCM and SCM modes were recorded by Raspberry Pi, and two primary datasets, one with CCM records and other with SCM records, were generated. The structure of logged data records in CCM and SCM are shown in Figure 8a,b, and the description of attributes is given in Table 1. For understanding, the sample data records logged during CCM are shown in Table 2, which includes the selections made by the child, such as preferred colors and illumination levels with durations. These interactions are logged with specific attributes. The date and time attribute records the exact moment of the interaction. The color attribute is recorded using abbreviations, such as R for red, B for blue, G for green, Y for yellow, and W for white. The illumination level is categorized into two broad intensity ranges: LI (low illumination) for levels with generated illumination less than 100 lux and HI (high illumination) for levels that generate illumination greater than 100 lux. These levels represent the brightness of the sensory stimuli generated by BT. The duration of the interaction is recorded in seconds, indicating how many seconds of time the child chose particular stimuli. On the other hand, the SCM data records shown in Table 3 include the system-generated combinations of colors and illumination levels and corresponding emotional responses exhibited by the child. The emotional responses of joy, surprise, anger, fear, sadness, and neutrality are categorized into positive and negative groups. Positive responses include joy and surprise, where the child shows happiness or excitement. Negative responses include anger, where the child displays frustration or distress; fear, indicating anxiety or discomfort; sadness, where the child appears downcast or disinterested; and neutral, which, while sometimes neutral, is often treated as negative due to a lack of positive engagement. The emotional response to each stimulus is recorded as either P (positive) or N (negative), reflecting the child’s immediate reaction.

### 2.7. Data Mining and Classification

#### 2.7.1. Data Mining

A data mining algorithm was developed by adopting techniques from the classical Apriori algorithm to identify single-item sets (e.g., illumination levels) and two-item sets (e.g., illumination level, emotional response), along with their respective support values within the interaction records collected during the child control mode (CCM) and system control mode (SCM). Support is defined as the proportion of transactions in the dataset that contain a particular item set. This is calculated by counting the occurrences of a particular item across the dataset and dividing it by the total number of transactions. For example, if the high illumination level is selected 10 times out of 20 recorded interactions, the support for the high illumination level would be calculated as 50%. For a clearer understanding of support calculation from the dataset, we have provided a text-based pseudocode in Algorithm 1.

In the CCM mode, the algorithm analyzes single-item sets related to the child’s preferences for different illumination levels, calculates their corresponding supports, and records the total duration of these preferences. The support values for high and low illumination levels, along with the total preferred duration, are used to classify sensory sensitivities. The pseudocode for this process is detailed in Algorithm 2, where the algorithm iterates through the dataset to compute the support values and total durations for the high and low illumination levels. This helps identify the child’s specific preferences and sensitivities based on illumination.

In the SCM mode, the algorithm evaluates two-item sets, specifically the combinations of illumination levels and their associated emotional responses (positive or negative). The support for these combinations is calculated based on the recorded data, and this information is used to classify the child’s emotional responses to varying illumination conditions. The pseudocode for this is given in Algorithm 3, which explains how the algorithm counts the occurrences of each combination of illumination level and emotional response and then calculates the respective support values. These calculations allow for the identification of emotional responses linked to specific illumination settings, which are critical for understanding the child’s sensory and emotional sensitivities.

**Algorithm 1**: Pseudo code for finding frequent single-item sets and their supports.Input: Dataset (A matrix where rows represent transactions and columns represent items. Each cell has a value of 1    if the item is present in the transaction, otherwise 0) Output: Item_support (A dictionary containing the support values for each item)Begin: 1. num_transactions ← number of rows in dataset 2. item_support ← an empty dictionary 3. For each item in the dataset columns:  a. count ← Sum of all values in the column corresponding to the item  b. support ← count / num_transactions  c. item_support[item] ← support 4. Return item_support End

**Algorithm 2**: Pseudo code for support calculation of child’s preferences for different illumination levels in CCM. INPUT: D = {(L1, T1), (L2, T2), ..., (Ln, Tn)}     # Dataset with illumination levels (Li={HI,LI}) and durations (Ti) OUTPUT: Support_HighIllumination, Support_LowIllumination, Total_HI_Duration, Total_LI_Duration FUNCTION CCM(D):  Count_HI = 0          # Count of high illumination instances Count_LI = 0          # Count of low illumination instances Total_HI_Duration = 0   # Total duration for high illumination Total_LI_Duration = 0   # Total duration for low illumination n = LENGTH(D)         # Total number of records in the dataset  # Loop through each illumination level and duration in the dataset FOR each (Li, Ti) IN D:  IF Li = HI THEN # High illumination instance   Count_HI += 1   Total_HI_Duration += Ti  ELSE IF Li = LI THEN # Low illumination instance   Count_LI += 1   Total_LI_Duration += Ti  END IF END FOR  # Calculate the support for high and low illumination levels Support_HighIllumination = Count_HI / n # Proportion of high illumination in the dataset Support_LowIllumination = Count_LI / n # Proportion of low illumination in the dataset  RETURN Support_HighIllumination, Support_LowIllumination, Total_HI_Duration, Total_LI_Duration

**Algorithm 3**: Pseudo code for support calculation for combinations of illumination levels and their associated emotional responses in SCM. INPUT: D = {(L1, E1), (L2, E2), ..., (Ln, En)} # Dataset with illumination levels (Li={HI,LI}) and emotional responses (Ei) OUTPUT: Support_HI_P, Support_HI_N, Support_LI_P, Support_LI_NFUNCTION SCM(D): Count_HI_P = 0     # Count of positive emotional responses for high illumination Count_HI_N = 0     # Count of negative emotional responses for high illumination Count_LI_P = 0     # Count of positive emotional responses for low illumination Count_LI_N = 0     # Count of negative emotional responses for low illumination n = LENGTH(D)     # Total number of records in the dataset  # Loop through each illumination level and emotional response in the dataset FOR each (Li, Ei) IN D:  IF Li = HI THEN       # High illumination   IF Ei = P THEN      # Positive emotional response    Count_HI_P += 1   ELSE IF Ei = N THEN    # Negative emotional response    Count_HI_N += 1   END IF  ELSE IF Li = LI THEN    # Low illumination   IF Ei = P THEN      # Positive emotional response    Count_LI_P += 1   ELSE IF Ei = N THEN   # Negative emotional response    Count_LI_N += 1   END IF  END IF END FOR  # support calculation for emotional responses linked to illumination levels Support_HI_P = Count_HI_P / n    # Proportion of high illumination with positive responses Support_HI_N = Count_HI_N / n    # Proportion of high illumination with negative responses Support_LI_P = Count_LI_P / n    # Proportion of low illumination with positive responses Support_LI_N = Count_LI_N / n    # Proportion of low illumination with negative responses  RETURN Support_HI_P, Support_HI_N, Support_LI_P, Support_LI_N

#### 2.7.2. Classification of Sensitivities

The classification process involves categorizing the child’s sensory responses into hyper-sensitive, hypo-sensitive, or normal. From the literature, it is evident that hypo-sensitive children are typically drawn to intense light, showing fascination with bright lights and their reflections. This indicates that hypo-sensitive children prefer high illuminated stimuli for longer durations when compared to low illuminated stimuli. A hyper-sensitive child often avoid bright lights, and they may become frightened by high illumination. This states that the child reacts negatively to high-intensity stimuli, indicating that they can only tolerate or prefer low illumination levels. A child with normal sensitivity shows balanced or typical responses to various levels of illumination without extreme preferences or aversions.

To quantify these observations and classify each child’s sensitivity in both child control mode (CCM) and system control mode (SCM), we used percentage difference as a key metric. The percentage difference formula, outlined in Equation (1), allows us to measure the relative differences between two values, A and B. For better understanding, we explain how these metrics are utilized for classification in CCM and SCM. In CCM, for each child, algorithm first computes the relative percentage difference (HLS_Diff) between the high illumination support (HI_S) and low illumination support (LI_S), as well as the relative percentage difference between the preferred durations (HLD_Diff) for high illumination (HI_D) and low illumination (LI_D) using the formulas shown in Equations (2) and (3). These calculations allow us to determine the sensory preferences for illumination levels and the duration of exposure. In SCM, similar calculations were applied to measure the children’s emotional responses to high and low illumination. Here, we compared high illumination positive response (HI_PR) with low illumination positive response (LI_PR) and high illumination negative response (HI_NR) with low illumination negative response (LI_NR). After calculating the relative percentage differences, a threshold value of 40% was considered to assess the significance of these differences. The 40% threshold is considered significant because it represents a substantial difference that indicates a strong preference or aversion. This threshold is chosen to ensure that the classification captures distinct behavioral patterns rather than minor variations. Based on these established behavioral patterns and quantitative assessments, certain rules were generated to classify a child’s sensory sensitivities into hypo-sensitive, hyper-sensitive, or normal categories, which are detailed in Table 4.

(1) $$Percentage\_difference=\frac{\left|A-B\right|}{\frac{A+B}{2}}\times 100$$

(2) $$HLS\_Diff=\frac{\left|HI\_S-LI\_S\right|}{\frac{HI\_S+LI\_S}{2}}\times 100$$

(3) $$HLD\_Diff=\frac{\left|HI\_D-LI\_D\right|}{\frac{HI\_D+LI\_D}{2}}\times 100$$

## 3. Evaluation of IoT-LSAS in Interactive Environment

The proposed IoT-LSAS was tested at the Autism Center of Excellence to determine its effectiveness in assessing sensory sensitivities in children with ASD. The participant’s selection and the evaluation procedure are explained in the following sub-sections.

### 3.1. Participant Selection

Indian children assessed with ASD (*n* = 20) aged three to fifteen years from the Autism Center of Excellence were selected for testing the feasibility of the proposed system. Participant selection was based on the Indian Scale for Assessment of Autism (ISAA), a widely utilized diagnostic tool in India for assessing ASD. Further, the clinical practitioner’s observations of the child’s behavior using conventional methods and interviews with the parents provided valuable insights into determining the child’s light sensitivities (hypo-sensitivity, hyper-sensitivity, or normal). The practitioner’s classification report is used as a baseline for evaluating the efficiency of IoT-LSAS. Detailed participant characteristics and ISAA scores are presented in Table 5.

### 3.2. Procedure

**Figure 9:**
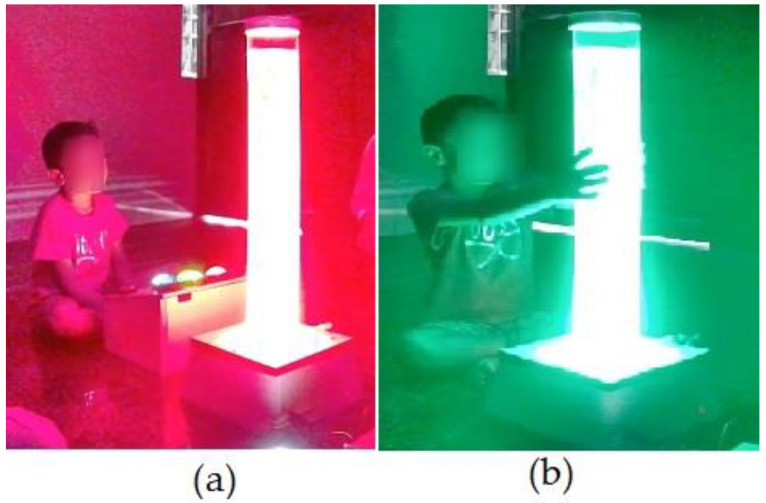
Child interaction with BT (**a**) CCM (**b**) SCM (the child’s face has been masked due to ethical concerns).

**Figure 10:**
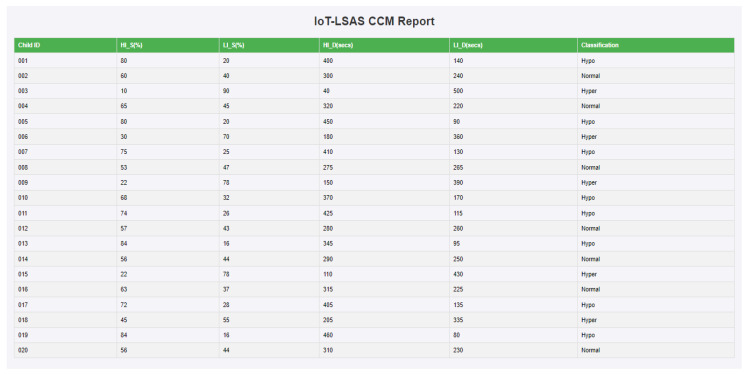
IoT-LSAS CCM report.

**Figure 11:**
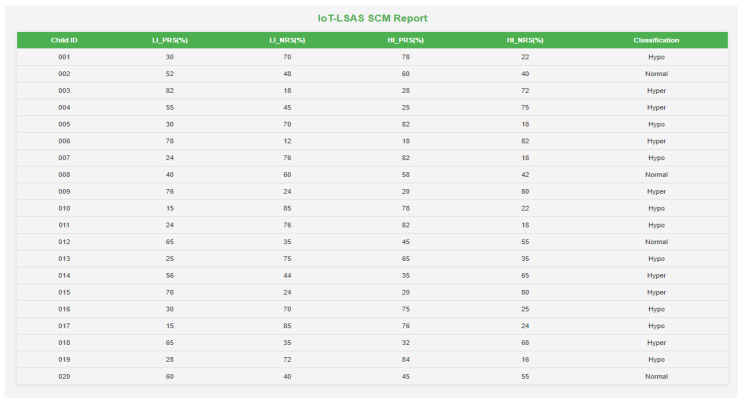
IoT LSAS SCM report.

A 10-foot by 10-foot observation room was exclusively allotted to carry out the study where the IoT-VSIS setup is placed. The observation room has enough space to keep the bubble tube setup and allow participants to have a comfortable distance to access the sensory stimuli from the bubble tube. This specific room is primarily designed to avoid stray sounds and minimize external distractions, creating a quiet and focused environment for the work. Initially, a trial run of a few sessions was conducted with volunteers to understand the placement of BT and to find out safe interaction distance. After consultation with volunteers and the therapist about the bubble tube placement and interaction distance, the child was instructed to sit half a meter away from the BT to ensure a comfortable distance for observation. No further instructions were given to avoid distractions that influence the child’s responses and allow for natural engagement. The child’s interaction with BT in CCM is shown in Figure 9a, whereas the child’s interaction in SCM is shown in Figure 9b. Each participating child engaged in a total of six sessions (three CCM sessions and three SCM sessions) each session lasted for 180 s. In each mode, the interactions of the child are logged by Raspberry Pi. From the logged data records, in CCM, the preferred colors, illumination levels, and the respective duration of interactions are calculated, and the classification report is generated, which is shown in Figure 10. In the generated report, the first column represents the child ID (identification number assigned to the child), the second column represents the support for high illumination preference (HI_S(%)), the third column represents the support for low illumination preference (LI_S(%), the fourth column represents the total duration for which the child interacted with high illumination stimuli (HI_D(secs)), the fifth column represents the total duration for which the child interacted with low illumination stimuli (LI_D(secs), and the last column is the classification based on support and interacted duration. Similarly, the report generated for SCM mode is shown in Figure 11, where the first column represents the child ID, the second column represents the support value of positive emotional reactions for low illumination levels (LI_PRS (%)), the third column represents the support value of negative emotional responses for low illumination levels (LI_NRS(%)), the fourth and fifth columns represent the support values of positive and negative emotional responses for high illumination levels (HI_PRS(%), HI_NRS(%)), and the last column shows the classification report generated based on analysis of emotional responses exhibited for high and low illumination levels.

The evaluation of the proposed IoT-LSAS system was conducted by comparing the classification outcomes from CCM and SCM with the practitioner classification reports shown in Table 6. The CCM classification report achieved a high agreement rate of 95% with the practitioner classification report. This indicates that the CCM is highly reliable in identifying sensory sensitivities, closely matching the assessment made using the traditional video coding method. The high agreement rate suggests that the IoT-LSAS system with CCM can accurately replicate traditional assessment outcomes in most cases. However, there was one notable mismatch where a child was classified as hypo-sensitive by the CCM mode, whereas traditional methods identified the child as having normal sensitivity. This discrepancy highlights an area where further refinement of the CCM mode may be needed to enhance accuracy. In contrast, the SCM mode, which focuses on emotional response tracking, had a 90% agreement rate with practitioner reports. A particular instance of mismatch in the SCM mode occurred when two children were classified as normal sensitive by the SCM and reported as hyper-sensitive in the practitioner’s report.

## 4. Discussion

It was observed from the evaluation results that IoT-LSAS could work effectively for assessing light sensitivities, which are either over-stimulating or under-stimulating to the child. A key feature of IoT-LSAS is its automated monitoring and analysis system, where individual monitoring reports are stored on the server, allowing clinical practitioners to view the child’s preferences and sensitivity reports through a user interface. However, there are some challenges and limitations to consider, particularly in SCM, where emotional response monitoring relies on the accuracy of facial expression recognition. Although it is an advanced method, there are possibilities for errors due to individual differences in expression and lighting conditions during assessments. One important limitation is that it is very difficult to classify facial expressions, especially in some cases involving children with ASD, who may not show clear emotional cues. Additionally, changes in lighting during the experiment can make it harder for the facial recognition system to accurately detect emotions. Moreover, differences in each child’s facial features and emotional expressions can lead to mistakes in identifying their feelings, especially if they do not match common expressions. To address these limitations, we could improve image preprocessing techniques by normalizing lighting conditions across frames, which would ensure more consistent facial feature detection. These models could be trained on larger and more diverse datasets, specifically tailored to children with ASD, to improve their accuracy. Using supplementary sensors for physiological measures (e.g., heart rate or skin conductance) could provide complementary emotional data helpful for accurate analysis. In contrast, CCM, which uses a stimuli preference method, has been found to be highly effective and accurate for diagnosing light sensitivities. Most practitioners who tested IoT-LSAS suggested that the CCM method is more effective due to its direct assessment of children’s preferences for various illumination levels and durations. This approach closely aligns with traditional coding schemes. Thus, the authors believe that IoT-LSAS with CCM can be used as an alternative to the manual video coding method for classifying light sensitivities while significantly reducing the time and effort of clinical practitioners. This was a pilot study with the primary goal of testing the system’s feasibility. The authors acknowledge the small sample size of 20 children as a limitation, and this may limit the generalizability of the results. Moreover, the current findings highlight the need for further validation studies involving larger, more diverse populations to enhance the reliability of the results. Future research could explore the integration of additional sensory modalities, such as auditory and tactile stimuli, to provide a more inclusive understanding of sensory sensitivities in children with ASD. By expanding the capabilities of the IoT-LSAS, authors believe that it can significantly enhance the assessment and understanding of sensory sensitivities not only in children with ASD but also in other groups facing similar sensory processing issues. Integrating auditory and tactile stimuli would provide a more comprehensive evaluation of sensory needs, allowing practitioners to develop tailored interventions based on each child’s individual sensory profile. This expanded system could lead to better treatment outcomes by enabling clinicians to design personalized sensory experiences that align more closely with each child’s unique sensitivities and preferences.

## 5. Conclusions

In this study, an IoT-LSAS was proposed and developed to identify light sensitivities in children with ASD. Using IoT, data logging, and analysis, the system provides an automated way to assess hyper- and hypo-sensitivities to light (color and illumination). The proposed system was tested on 20 children, and the system showed a 95% agreement with practitioner assessments in child control mode and 90% in system control mode. The results validate that IoT-LSAS performs well enough, and it can be considered a substitute for conventional assessment techniques in diagnosing light sensitivities in children with ASD. Further research and development could expand the system’s capabilities in detecting sensitivities related to other sensory systems, such as auditory and tactile responses.

## Figures and Tables

**Table 1:** Description of attributes.

Attribute	Description
Date and Time	Timestamp of the sensory interaction event, formatted as YYYY-MM-DD HH:MM
Color	Abbreviation representing the color (R for red, B for blue, G for green, Y for yellow and W for white)
Illumination level	Level of intensity measured in lux, low illumination (LI) to high illumination (HI)
Duration	Duration of the sensory interaction in seconds
Emotional Response	Abbreviation for emotional response category (e.g., P for positive, N for negative emotional responses)

**Table 2:** Sample data records logged during CCM.

Date and Time	Color	Illumination Level	Duration (s)
2024-07-23 14:35:10	B	HI	60
2024-07-23 14:36:50	R	LI	40
2024-07-23 14:38:15	G	LI	30

**Table 3:** Sample data records logged during SCM.

Date and Time	Color	Illumination Level	Emotional Response
2024-07-23 14:35:10	B	HI	P
2024-07-23 14:36:50	R	LI	N
2024-07-23 14:38:15	G	LI	N

**Table 4:** Rules for classifying light sensitivities in CCM and SCM.

Classification	Rule (CCM Mode)	Rule (SCM Mode)
Hypo-Sensitive	If the child shows a significantly greater preference for high illumination levels with long duration compared to low illumination levels (HLS_Diff ≥ 40% and D_Diff ≥ 40%, with HI_S > LI_S and HI_D > LI_D).	If high illumination positive response (HI_PR) is significantly higher than low illumination positive response (LI_PR) and high illumination negative response (HI_NR) is significantly lower than low illumination negative response (LI_NR) with a threshold of ≥40% difference.
Hyper-Sensitive	If the child consistently shows a significant preference for low illumination levels with long duration compared to high illumination levels (HLS_Diff ≥ 40% and D_Diff ≥ 40%, with LI_S > HI_S and LI_D > HI_D).	If low illumination positive response (LI_PR) is significantly higher than high illumination positive response (HI_PR) and low illumination negative response (LI_NR) is significantly lower than high illumination negative response (HI_NR) with a threshold of ≥40% difference.
Normal	If there is no significant difference in preference for high versus low illumination levels, or if preferences are balanced across different intensities and durations (HLS_Diff < 40% and D_Diff < 40%).	If the child shows balanced emotional responses regardless of whether the illumination level is high or low, the child is classified as normal.

**Table 5:** Participant characteristics and ISAA scores. (y-years and m-months).

Child ID	Age	ISAA Score	Autism Classification
001	9y	103	Mild
002	4y 1m	106	Mild
003	4y	120	Moderate
004	5y 9m	89	Mild
005	6y	131	Moderate
006	9y	92	Mild
007	4y 2m	127	Moderate
008	7y 1m	116	Moderate
009	6y 2m	108	Moderate
010	5y 3m	128	Moderate
011	8y	121	Moderate
012	7y 3m	100	Mild
013	4y 2m	106	Mild
014	5y 6m	87	Mild
015	4y 8m	102	Mild
016	6y 3m	134	Moderate
017	8y 2m	118	Moderate
018	7y 5m	108	Moderate
019	5y 2m	126	Moderate
020	6y 4m	97	Mild

Child ID

Age

ISAA Score

Autism Classification

**Table 6:** Child sensory sensitivity classifications from practitioners, CCM, and SCM.

Child ID	Practitioners Classification Report	CCM Classification Report	SCM Classification Report
001	Hypo-sensitive	Hypo-sensitive	Hypo-sensitive
002	Normal	normal	normal
003	Hyper-sensitive	Hyper-sensitive	Hyper-sensitive
004	Normal	Normal	Hyper-sensitive
005	Hypo-sensitive	Hypo-sensitive	Hypo-sensitive
006	Hyper-sensitive	Hyper-sensitive	Hyper-sensitive
007	Hypo-sensitive	Hypo-sensitive	Hypo-sensitive
008	Normal	Normal	Normal
009	Hyper-sensitive	Hyper-sensitive	Hyper-sensitive
010	Hypo-sensitive	Hypo-sensitive	Hypo-sensitive
011	Hypo-sensitive	Hypo-sensitive	Hypo-sensitive
012	Normal	Normal	Normal
013	Hypo-sensitive	Hypo-sensitive	Hypo-sensitive
014	Normal	Normal	Hyper-sensitive
015	Hyper-sensitive	Hyper-sensitive	Hyper-sensitive
016	Hypo-Sensitive	Normal	Hypo-sensitive
017	Hypo-sensitive	Hypo-sensitive	Hypo-sensitive
018	Hyper-sensitive	Hyper-sensitive	Hyper-sensitive
019	Hypo-sensitive	Hypo-sensitive	Hypo-sensitive
020	Normal	Normal	Normal

## Data Availability

The data analyzed during the current study are available upon reasonable request from the corresponding author.

## References

[1] (2013). Diagnostic and Statistical Manual of Mental Disorders.

[2] McCormick C., Hepburn S., Young G.S., Rogers S.J. (2016). Sensory Symptoms in Children with Autism Spectrum Disorder, Other Developmental Disorders and Typical Development: A Longitudinal Study. Autism.

[3] Lord C., Brugha T.S., Charman T., Cusack J., Dumas G., Frazier T., Jones E.J., Jones R.M., Pickles A., State M.W. (2020). Autism Spectrum Disorder. Nat. Rev. Dis. Primers.

[4] Dunn W. (1997). The Impact of Sensory Processing Abilities on the Daily Lives of Young Children and Their Families: A Conceptual Model. Infants Young Child..

[5] Tomchek S.D., Dunn W. (2007). Sensory Processing in Children with and without Autism: A Comparative Study Using the Short Sensory Profile. Am. J. Occup. Ther..

[6] Chang Y.-S., Owen J.P., Desai S.S., Hill S.S., Arnett A.B., Harris J., Marco E.J., Mukherjee P. (2014). Autism and Sensory Processing Disorders: Shared White Matter Disruption in Sensory Pathways but Divergent Connectivity in Social-Emotional Pathways. PLoS ONE.

[7] Cheung P.P.P., Siu A.M.H. (2009). A Comparison of Patterns of Sensory Processing in Children with and without Developmental Disabilities. Res. Dev. Disabil..

[8] Coulter R.A. (2009). Understanding the Visual Symptoms of Individuals with Autism Spectrum Disorder (ASD). Optom. Vis. Dev..

[9] Raucci U., Di Nardo G., Evangelisti M., Villa M.P., Parisi P. (2021). Photosensitivity in Various Disease States. The Importance of Photosensitivity for Epilepsy.

[10] Marco E.J., Hinkley L.B.N., Hill S.S., Nagarajan S.S. (2011). Sensory Processing in Autism: A Review of Neurophysiologic Findings. Pediatr. Res..

[11] Geilman A. (2016). Designing for Children with Sensory Integration Disorders: A Handbook for Residential Designers. JCCC Honor. J..

[12] Bogdashina O. (2016). Sensory Perceptual Issues in Autism and Asperger Syndrome: Different Sensory Experiences-Different Perceptual Worlds.

[13] Baranek G.T., David F.J., Poe M.D., Stone W.L., Watson L.R. (2006). Sensory Experiences Questionnaire: Discriminating Sensory Features in Young Children with Autism, Developmental Delays, and Typical Development. J. Child Psychol. Psychiatry.

[14] Schulz S.E., Stevenson R.A. (2020). Differentiating between Sensory Sensitivity and Sensory Reactivity in Relation to Restricted Interests and Repetitive Behaviours. Autism.

[15] Franklin A., Sowden P., Burley R., Notman L., Alder E. (2008). Color Perception in Children with Autism. J. Autism Dev. Disord..

[16] Ludlow A., Heaton P., Hill E., Franklin A. (2014). Color obsessions and phobias in autism spectrum disorders: The case of J.G. Neurocase.

[17] Unwin K.L., Powell G., Jones C.R. (2022). The Use of Multi-Sensory Environments with Autistic Children: Exploring the Effect of Having Control of Sensory Changes. Autism.

[18] Slevin E., Mcclelland A. (1999). Multisensory Environments: Are They Therapeutic? A Single-Subject Evaluation of the Clinical Effectiveness of a Multisensory Environment. J. Clin. Nurs..

[19] Atari R. (2014). The Influence of Multi-Sensory Environment on Physiological Response in Children with Autism Spectrum Disorders and Children with Special Health Care Needs.

[20] Haegele J.A., Porretta D.L. (2014). Snoezelen Multisensory Environment. Palaestra.

[21] Riederer M., Schoenauer C., Kaufmann H., Soechting E., Lamm C. Development of Tests to Evaluate the Sensory Abilities of Children with Autism Spectrum Disorder Using Touch and Force Sensors. Proceedings of the 4th International Conference on Wireless Mobile Communication and Healthcare-Transforming Healthcare Through Innovations in Mobile and Wireless Technologies (MOBIHEALTH).

[22] Brown C., Stein F. (2024). Adolescent/Adult Sensory Profile. Assessments in Occupational Therapy Mental Health.

[23] Hailpern J., Karahalios K., Halle J., DeThorne L., Coletto M.K. A3: A Coding Guideline for HCI+ Autism Research Using Video Annotation. Proceedings of the 10th International ACM SIGACCESS Conference on Computers and Accessibility.

[24] Clifford S., Young R., Williamson P. (2007). Assessing the Early Characteristics of Autistic Disorder Using Video Analysis. J. Autism Dev. Disord..

[25] Söchting E., Hartl J., Riederer M., Schönauer C., Kaufmann H., Lamm C. (2015). Development of Tests to Evaluate the Sensory Abilities of Children with Autism Spectrum Disorder. Procedia Comput. Sci..

[26] Tang F., McMahan R.P., Allen T.T. Development of a Low-Cost Tactile Sleeve for Autism Intervention. Proceedings of the IEEE International Symposium on Haptic, Audio and Visual Environments and Games (HAVE).

[27] Khullar V., Singh H.P., Bala M. (2019). IoT Based Assistive Companion for Hypersensitive Individuals (ACHI) with Autism Spectrum Disorder. Asian J. Psychiatry.

[28] Yu T., Park K.W., McKeown M.J., Wang Z.J. (2023). Clinically Informed Automated Assessment of Finger Tapping Videos in Parkinson’s Disease. Sensors.

[29] Cabello-Collado C., Rodriguez-Juan J., Ortiz-Perez D., Garcia-Rodriguez J., Tomás D., Vizcaya-Moreno M.F. (2024). Automated Generation of Clinical Reports Using Sensing Technologies with Deep Learning Techniques. Sensors.

[30] Unwin K.L., Powell G., Price A., Jones C.R. (2024). Patterns of Equipment Use for Autistic Children in Multi-Sensory Environments: Time Spent with Sensory Equipment Varies by Sensory Profile and Intellectual Ability. Autism.

[31] Seckman A., Paine C.W., Gilmer L., Fisch G.S., Gulley J., Hodas G.R., McKinney C. (2017). Evaluation of the Use of a Sensory Room on an Adolescent Inpatient Unit and Its Impact on Restraint and Seclusion Prevention. J. Child Adolesc. Psychiatr. Nurs..

[32] Baillon S., van Diepen E., Prettyman R. (2002). Multi-Sensory Therapy in Psychiatric Care. Adv. Psychiatr. Treat..

[33] Lorusso L.N., Southwick S.M., Charney D.S., Rasmusson A.M. (2020). Sensory Environments for Behavioral Health in Dementia: Diffusion of an Environmental Innovation at the Veterans Health Administration. HERD.

[34] Gupta A., Kumar R., Khera A., Sankar S., Khurmi S., Srivastava M. (2013). Operational Guidelines Rashtriya Bal Swasthya Karyakram.

